# Assessing adverse events associated with chiropractic care in preschool pediatric population: a feasibility study

**DOI:** 10.1186/s12998-024-00529-0

**Published:** 2024-03-13

**Authors:** Anne Dolbec, Chantale Doucet, Katherine A Pohlman, Stéphane Sobczak, Isabelle Pagé

**Affiliations:** 1 Département d’anatomie, Université du Québec à Trois-Rivières, 3351 Boul. des Forges, G8Z 4M3 Trois-Rivières, Québec Canada; 2 Département de chiropratique, Université du Québec à Trois-Rivières, 3351 Boul. des Forges, G8Z 4M3 Trois-Rivières, Québec Canada; 3 Research Center, Parker University, 2540 Walnut Hill Lane, 75229 Dallas, TX USA; 4 Centre Interdisciplinaire de recherche en réadaptation et intégration sociale (Cirris), Centre Intégré Universitaire de Santé et de Services Sociaux de la Capitale-Nationale (CIUSS-CN), 525 Boul. Wilfrid-Hamel, G1M 2S8 Québec, Québec Canada; 5 Groupe de recherche sur les affections neuromusculosquelettiques (GRAN), Université du Québec à Trois-Rivières, 3351 Boul. des Forges, G8Z 4M3 Trois-Rivières, Québec Canada; 6 Chaire de recherche en anatomie fonctionnelle, Université du Québec à Trois-Rivières, 3351 Boul. des Forges, G8Z 4M3 Trois-Rivières, Québec Canada

**Keywords:** Manual therapies, Pediatrics, Spinal mobilizations, Spinal manipulations, Side effects, Safety

## Abstract

**Background:**

Manual therapies are commonly used by healthcare professionals when caring for children. However, few prospective studies have evaluated their adverse events (AEs). This study aims to assess the feasibility of a pragmatic prospective study aiming to report the immediate and delayed (48-hours post-treatment) AEs associated with manual therapies in children aged 5 or younger. Preliminary data on AEs frequency are also reported.

**Methods:**

Between July 2021 and March 2022, chiropractors were recruited through purposive sampling and via a dedicated Facebook group for Quebec chiropractors interested in pediatrics. Legal guardians of patients aged 5 or younger were invited to fill out an online information and consent form. AEs were collected using the SafetyNET reporting system, which had been previously translated by the research team. Immediate AEs were collected through a questionnaire filled out by the legal guardian immediately after the treatment, while delayed AEs were collected through a questionnaire sent by email to the legal guardian 48 h after the treatment. Feasibility was assessed qualitatively through feedback from chiropractors and quantitatively through recruitment data.

**Results:**

Overall, a total of 28 chiropractors expressed interest following the Facebook publication, and 5 participated. An additional two chiropractors were enrolled through purposive sampling. In total, 80 legal guardians consented to their child’s participation, and data from 73 children were included for the analysis of AEs. At least one AE was reported in 30% of children (22/73), and AEs were mainly observed immediately following the treatment (16/22). The most common AEs were irritability/crying (11 children) or fatigue/tiredness (11 children). Feasibility analysis demonstrated that regular communication between the research team and clinicians, as well as targeting clinicians who showed great interest in pediatrics, were key factors for successful research.

**Conclusion:**

Results suggest that it is feasible to conduct a prospective pragmatic study evaluating AEs associated with manual therapies in private practices. Direct communication with the clinicians, a strategic clinicians’ recruitment plan, and the resulting administrative burden should be considered in future studies. A larger study is required to confirm the frequency of AEs reported in the current study.

**Trial registration:**

ClinicalTrials.gov., NCT05409859, Registered on June 3 2022. https://clinicaltrials.gov/study/NCT05409859.

**Supplementary Information:**

The online version contains supplementary material available at 10.1186/s12998-024-00529-0.

## Background

Since the last decade, an increasing number of parents are consulting complementary and alternative medicine (CAM) practitioners for their children’s health-related disorders [1]. A 2014 systematic review from Italyreported that prevalence rates for overall CAM use for children (i.e., under the age of 17 years) ranged from 10.9 to 87.6% for a lifetime use and from 8 to 48.5% for current use, based on 58 eligible studies from 19 countries [2]. Among modalities provided by CAM practitioners, manual therapies, including both spinal manipulation and mobilization, appear to have an important role in the healthcare treatment of neuromusculoskeletal disorders and are regulated in many countries [3]. Spinal manipulation is defined as the application of a force to a spinal joint using a high velocity, while spinal mobilization is defined as the application of a cyclic force using a slow velocity and is considered the most appropriate technique for young children [4].

Among CAM practitioners, chiropractors are one of the most common professions to consistently use manual therapies for the management of pediatric musculoskeletal disorders [5]. Moreover, pediatric patients (i.e., under the age of 17 years) has been reported to constitute up to 38.7% of the patient population of chiropractors specializing in pediatrics [5]. Musculoskeletal complaints, such as back pain and neck pain, have been reported as the main reasons to receive manual therapies, but pediatric patients may present with a variety of conditions, such as attention deficit/hyperactivity disorder (ADHD), asthma, wellness/prevention, breastfeeding problems, neck torticollis, and plagiocephaly [6, 7]. It is important to note that the management by the practitioner will not necessarily target the treatment of these conditions, but the neuromusculoskeletal symptoms that may be associated with them.

Despite the popularity of manual therapies in the pediatric population, the safety of spinal mobilization in children is poorly understood [8]. Systematic reviews of the literature (e.g., Todd et al. [9] and Vohra et al. [10]) revealed that while adverse events (AEs) are rare, they can occur. Reported AEs are mostly mild and self-limiting with the most common being increased irritability or crying and discomfort or pain [9]. However, while serious AEs have been reported, their incidence is infrequent and is often linked to pre-existing pathologies affecting the neuromusculoskeletal system. For instance, in a documented case report, a 4-month-old infant experienced quadriplegia following manual therapy, with a direct association to the presence of an undiagnosed spinal cord astrocytoma [11]. Another case highlights adverse events associated with the use of inappropriate techniques or their improper application by practitioners, such as the unfortunate incident where persistent forced neck and spine flexion during a craniosacral technique led to the tragic death of a 3-month-old infant [12]. Recently, a cluster randomized controlled trial in which AEs were evaluated by the clinician and by the parents or legal guardians of children seeking care in chiropractic within the USA and Canada has been conducted [6]. Overall, AEs were reported in 8.8% of the chiropractic visits with children (less than 14 years of age) with no serious AE observed. To our knowledge, this study constitutes the first high quality prospective study aiming specifically to report AEs following manual therapies in children. Consequently, further studies are necessary to gather conclusive data, enhance clinical management of the pediatric population, and determine effective strategies for implementing such clinical projects in various healthcare settings.

Therefore, the main objective of study is to evaluate the feasibility to conduct a pragmatic prospective study aiming to report the frequency of immediate and delayed (48 h post-treatment) AEs associated with manual therapies in children of 5 years or less. The secondary objective is to report preliminary data on AEs frequency, their nature (i.e. new symptoms or worsening of previously reported symptoms), as well as to compare age, sex and treated region(s) between those who sustained an AE and those who didn’t. Based on the results of Paanalahti and al [13], it was hypothesized that the occurrence of an AE will be more frequent in female participants and in those only treated at the cervical region [13]. We also hypothesized that AE frequency will be higher in older children considering that it might be easier for the legal guardian to observe an AE in older pre-school children than in neonates or infants.

## Methods

### Study design

The study design is an observational and pragmatic prospective cohort study including both a descriptive and analytical portion. The protocol was developed in line with the Strengthening the Reporting of Observational Studies in Epidemiology (STROBE) guidelines for reporting observational cohort studies [14]. This study was conducted in accordance with the University Research Ethics Board (CER-21-278-07.05) and was prospectively registered on ClinicalTrials.gov (NCT05409859). Furthermore, adherence to the CONSORT feasibility checklist for intervention studies and compliance with the STROBE checklist for observational studies were ensured.

### Study overview

**Fig. 1:**
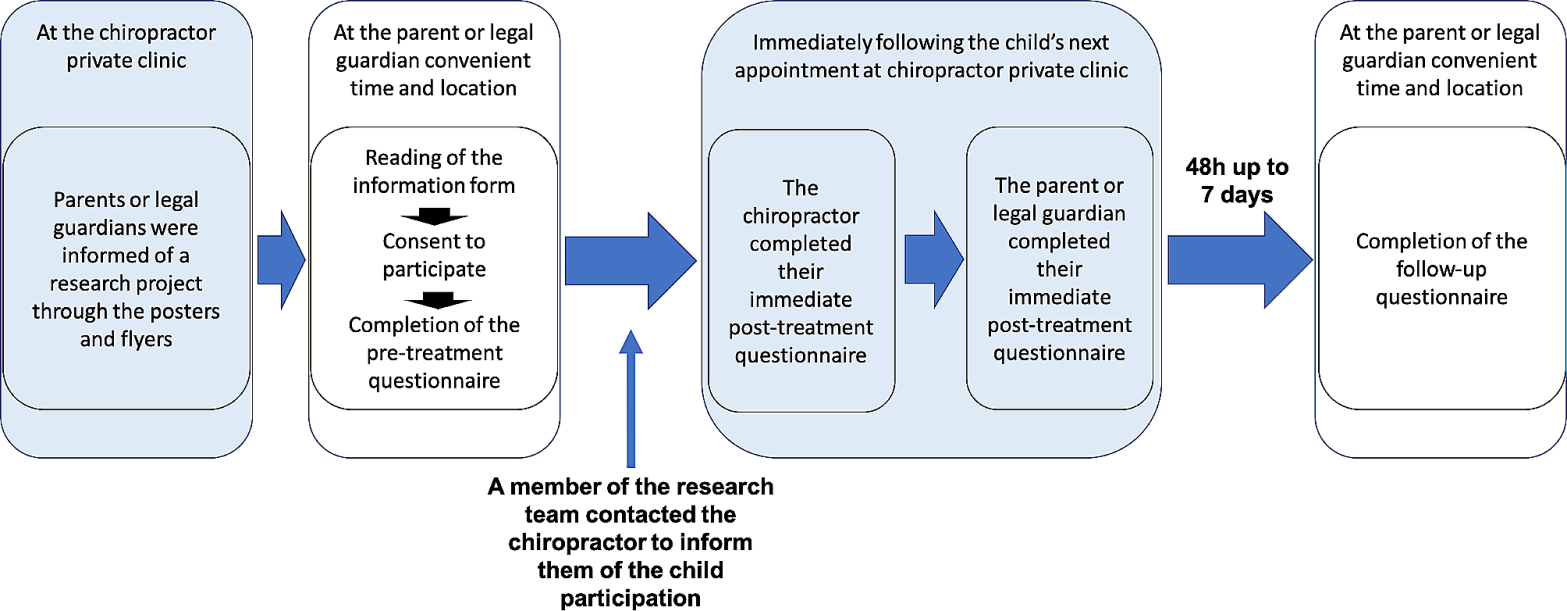
Overview of the study procedures

Figure 1 presents an overview of the study procedures. Clinicians (chiropractors) from Quebec Province (Canada) were invited into the research project through a Facebook post on a private group and by purposive sampling method of recruitment through direct communications with chiropractors known by the investigative team to treat pediatric patients. Only the first five clinicians contacting the research team and meeting eligibility criteria were invited to take part in the study. Parents or legal guardians (further referred to as legal guardians) of children aged 5 years or less who were seeking care from the participating clinicians were invited to participate in this study. There was information on flyers and posters at the private clinics of participating clinicians to inform the legal guardian. AEs were subsequently collected through two questionnaires completed by the legal guardian, one immediately following the next appointment of the child and the other 48 h after that appointment. Clinicians also completed a questionnaire immediately following the next appointment to describe the treatment provided to the child. All questionnaires were available online and have been used in previous SafetyNET reporting system studies (e.g. Pohlman et al. 2014 [15], 2020 [6], 2020 [16]). Each procedure is detailed below.

### Clinicians’ eligibility criteria and recruitment

To participate in the study, clinicians had to practice in a chiropractic private clinic and had to report interest and capacity in caring for the pediatric population during the study data collection time period. In July 2021, for feasibility purposes, data collection was initiated with two clinicians who were recruited through direct communication. They were instructed to identify any issues with the study protocol. Ten weeks later, and continuing until March 2022, additional clinicians were recruited through an advertisement posted in a private Facebook group for chiropractors with a specific interest in pediatric care. Interested clinicians contacted the research team by email to schedule a meeting during which the study procedures were explained to ensure standardization. While the study focused on evaluating AEs associated with manual therapy, clinicians were instructed to administer their standard treatment protocols, which could include spinal mobilization or other interventions typically provided to patients. Clinicians were also invited to contact the research team at any time during the study if they had questions. Due to the administrative burden of the study, consent was provided by the clinicians following the meeting if they remained interested and had their questions answered. Participation of each clinician was also started at different time points. Before starting their participation, a package containing an electronic tablet, posters and flyers was sent to the clinician’s private clinic. Posters and flyers included a brief description of the study, the criteria for the children to participate in the study, the email to contact the research team, the ethic certification number and a QR code to access the information sheet for ease of use by potential patients and legal guardians. Clinicians were prompted to engage in patient recruitment until they chose to withdraw (with an invitation to articulate their reasons) or until the research team concluded their participation, facilitating the inclusion of new clinicians. This was implemented to maintain a limited number of clinicians actively participating simultaneously. Data collection ended in March 2022.

### Children’s eligibility criteria and recruitment

Immediately following the information sheet accessed with the QR code, the legal guardian could review the consent for their child. The legal guardian could review the information sheet and consent form after they left the clinician’s private clinic to ensure full comprehension and not to feel any pressure to participate. The eligibility criteria were that the child was aged 5 years or less and was consulting with one of the participating clinicians. To be included, the appointment considered for the AE collection had to be the child’s first or second treatment. Before consenting to the child’s participation, the legal guardian could ask to be contacted by a member of the research team to answer any questions or concerns. When a consent form was signed, the research team then immediately contacted the child’s clinician to notify them of the child’s participation.

### Sample size

The sample size of clinicians was determined based on the research team’s capacity for adequate follow-up, with a targeted minimum sample of five clinician participants. A limit of four clinicians actively participating at the same time was set. No specific sample size was targeted for child participants. Clinicians were invited to recruit child patients until the end of their active participation or the conclusion of the data collection period, which was March 2022.

### SafetyNET reporting system

The Canadian French pediatric version of the SafetyNET reporting system was used to gather the AEs (under redaction). This reporting system has been shown to be valid to report AEs associated with manual therapies [15] and is managed using the Research Electronic Data Capture internet (REDCap) platform [17, 18]. Relevant parts of the reporting system used in the current study are detailed below.

#### Pre-treatment questionnaire completed by the legal guardian

Immediately following the consent, the legal guardian completed a questionnaire to gather demographic information (child sex and date of birth), as well as information in regards of the reasons to seek care in chiropractic and the symptom(s) presented by the child. Reason(s) to seek care was collected using a check list and included: preventative/wellness/no symptoms, headache/migraines, neck pain, mid-back or rib pain, low-back pain, extremities pain, attention deficit disorder/attention deficit/hyperactivity disorder (ADD/ADHD), autism, breastfeeding difficulties, cold, colic, digestive issues, plagiocephaly, torticollis, and other reasons. Symptom(s) presented by the child was similarly gathered and included: none, discomfort/pain, stiffness, weakness, fatigue/tiredness, headache, dizziness, numbness/tingling, nausea/vomiting, difficulty walking, problems sleeping, irritability/crying, and other symptom(s). If “other reason(s)” or “other symptom(s)” was checked, the other reason(s) or symptom(s) had to be specified.

#### Immediate post-treatment questionnaire completed by the clinician

Immediately following the treatment and using the electronic tablet provided by the research team, the clinician completed a questionnaire to describe the treatment provided to the child. The questionnaire consisted of a checklist to indicate all of the body part(s) treated by the clinician: cervical, thoracic, lumbar/pelvis, upper extremity, and lower extremity. The treatment modality(ies) was also gathered: manipulation, mobilization, use of a mechanical device, other manual therapy, and other non-manual therapy.

#### Immediate post-treatment questionnaire completed by the legal guardian

Once the clinician completed the immediate post-treatment questionnaire, they handed the electronic tablet to the legal guardian for completing their immediate post-treatment questionnaire. The legal guardians were instructed to check all the symptom(s) observed or expressed by their child immediately following the treatment. The potential symptoms were the same list from the pre-treatment questionnaire.

#### Follow-up questionnaire

A follow-up questionnaire was sent to the email address provided by the legal guardian 48 h following the appointment. If the questionnaire was not completed at that time, it was sent again every 24 h until completion or for a maximum of 6 email attempts. The legal guardian was asked to evaluate the evolution (worsening, no change, or improvement) of symptom(s). Finally, the legal guardian had to report any new symptom(s) observed since the appointment, which was the same list from the pre-treatment questionnaire.

### Feasibility analysis

With the first two participating clinicians, frequent follow-ups were planned to collect their feedback on the study, including any study procedure changes to help improve data collection procedures. If possible, changes were implemented before starting data collection with the other clinicians. Feasibility was also determined quantitatively through recruitment efficiency. A descriptive analysis of the number of clinicians who viewed the Facebook advertisement, who expressed their interest to participate, who consented to participate, and who actually participated in the study was computed, as well as the number of legal guardians who read the information sheet, who consented to participate, and who completed each of the three questionnaires.

### AEs analysis

AEs analysis included participants who had an immediate post-treatment questionnaire and/or the follow-up questionnaire completed. A descriptive analysis of the children demographics (age and sex) and initial characteristics (reasons to seek care and pre-treatment symptoms) was first undergone. The nature of AEs experienced by the children was subsequently analyzed at the two time points, i.e., immediate AEs and delayed AEs. AEs were defined as either a new symptom or the worsening of pre-existing symptoms, as self-reported by the legal guardian [15]. The frequency of AEs at both time points was then calculated by dividing the number of questionnaires in which at least one AE symptom was reported by the total number of available questionnaires. A Pearson’s chi-square test was computed to compare the proportion of female and male children for which an AE was reported. Difference in the age of the children reporting an AE or not was evaluated using a Mann-Whitney U test. Although it was initially planned to evaluate whether the treated region(s) influenced the occurrence of an AE, this analysis was not possible because > 90% of the children were treated at multiple regions. SPSS 26.0 (Armonk, NY: IBM Corp) was used to analyze the data and significance was set at *p* <.05.

If any serious AEs were identified, they would be reported per normal regulations per ethical requirements. Serious AEs were defined according to Pohlman et al. [15] as an AE resulting in death or is life threatening or results in inpatient hospitalization or prolongation of existing hospitalization for more than 24 h with a persistent or significant incapacity or substantial disruption of the ability to conduct normal life functions.

## Results

### Clinicians recruitment and initial characteristics

A total of 7 clinicians participated in the study. From the 112 clinicians who viewed the Facebook post, 25% (28/112) expressed interest in participating in the study. The first five clinicians were invited to participate in the study in addition to the two clinicians who were recruited by purposive sampling method. All clinicians (*n* = 7; 100% women) provided their consent to participate and graduated from the Université du Québec à Trois-Rivières (UQTR) chiropractic program (Canada) between 2000 and 2019. Moreover, 71.4% (5/7) detained a Diplomate in Clinical Chiropractic Pediatrics (DICCP) from the International Chiropractors Association (ICA), which is a 3-year program focused on the clinical management of pregnant women, babies, and children. Participants were age 38.4 ± 8 years (mean ± SD; range, 32–52 years old) and were in private practice for 12.7 ± 6.9 years (mean ± SD; range 3–22). One clinician terminated their participation after two weeks of active patient recruitment, citing the additional time required for each treatment session with a participating child. Other clinicians continued their involvement until the research team concluded their participation, either to facilitate the inclusion of new clinicians or until the conclusion of data collection in March 2022.

### Feasibility and implemented changes

A follow-up was done with the first two clinicians every two weeks throughout their data collection. Overall, two main challenges were identified: complexity with patient consent to participation before treatment; time burden for the clinician and their clinic staff. Clinicians reported that several legal guardians would have liked to provide consent to participate at the clinic before or immediately after the appointment instead of completing the form outside the clinic and having to wait for the next appointment for the data collection. Due to ethics requirements, legal guardians had to consent and complete the pre-treatment questionnaire before the appointment, but whenever possible, clinicians delayed the treatment to provide time to the legal guardian to read and complete the online forms. As a result, clinicians mentioned that they were falling behind. Clinicians also highlighted that, although the legal guardians were invited to directly contact the research team for any inquiries, they often asked them questions or were excited to talk about the project. Five to ten minutes had to be added to the regular appointment duration due to discussions with the legal guardian and time to complete the immediate post-treatment questionnaire. The research team informed the other participating clinicians of the extra time needed so that they could adapt their schedule and to brief their clinic staff about the study and instruct them to direct legal guardians toward the research team for any questions.

Besides these comments, the first two clinicians provided an overall positive feedback regarding the study protocol. They reported that the initial meeting with a researcher was very helpful to understand the study procedures. They also appreciated receiving flyers and posters, as well as a sheet summarizing their tasks that they could keep within the treatment rooms. Not having to use their own laptop or cell phone to complete the immediate questionnaires was also appreciated and made the study easier to conduct. Clinicians also valued being able to contact a member of the research team at any time, as well as the frequent follow-ups by the researcher to ensure everything was going smoothly.

### Children recruitment and study flow

**Fig. 2:**
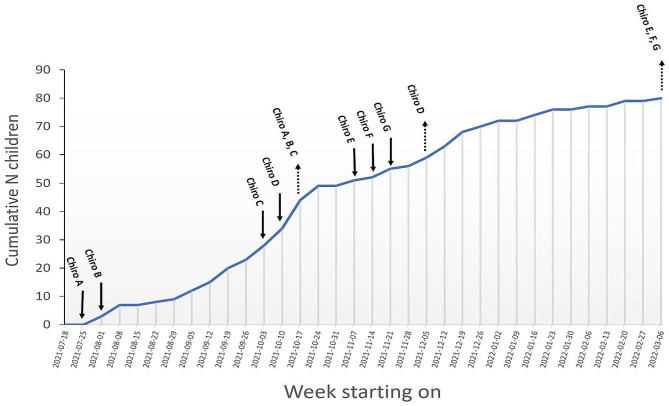
Recruitment graph. Time points at which clinicians started (full arrows) and ended (dotted arrows) their active participation, along with the cumulative number of children for which legal guardian consented to their participation throughout the active data collection period

**Fig. 3:**
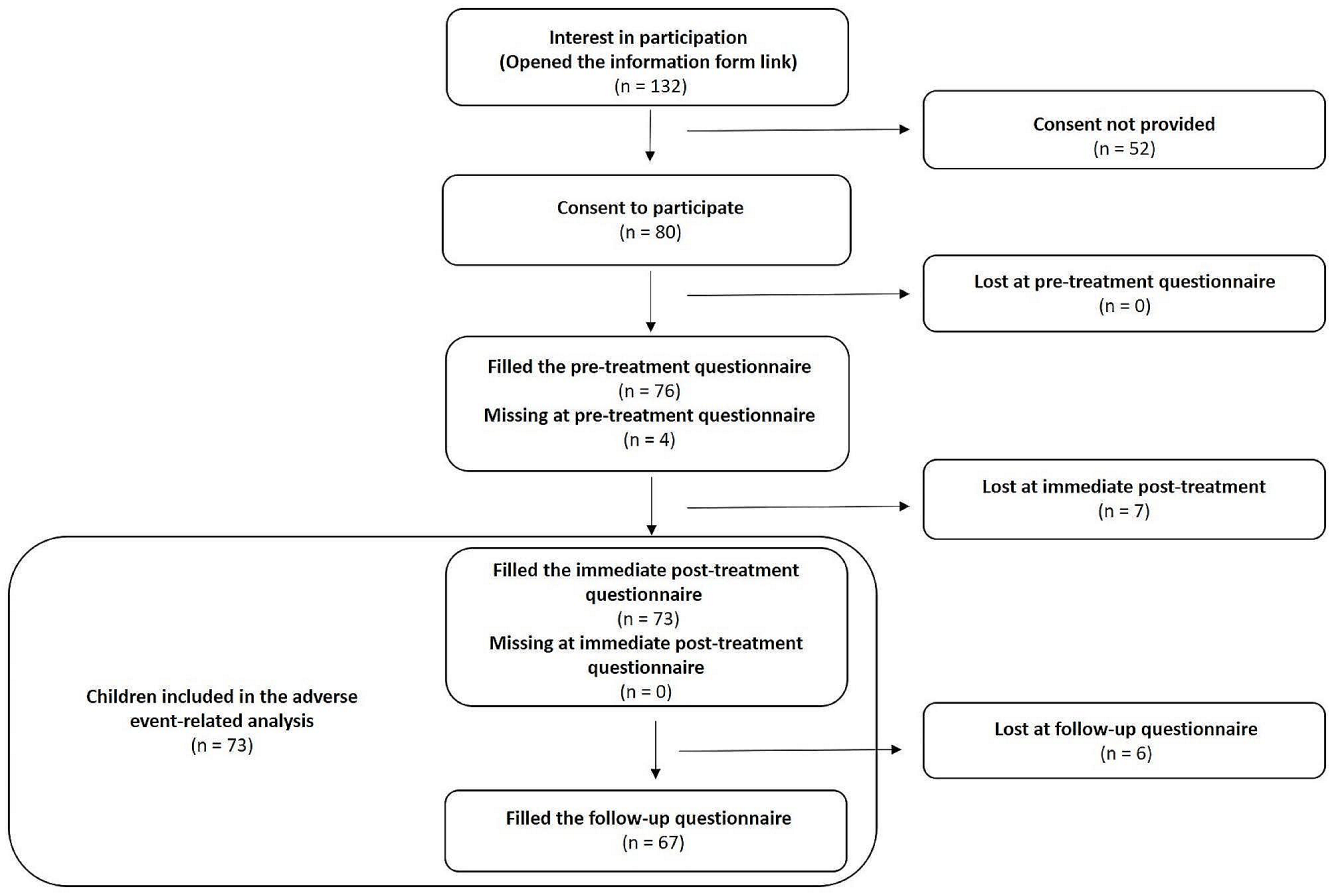
Study flow chart of participating children. Note that the absence of a pre-treatment questionnaire or an immediate post-treatment questionnaire did not preclude legal guardians from participating in the remaining aspects of the study

Figure 2 outlines the number of legal guardians who provided their consent to the participation of their child throughout the data collection period for all participating clinicians. Figure 3 outlines the participation of the patients. From the 132 legal guardians who accessed the information form using the web link, 60.6% (80/132) consented to the participation of their child. AEs analysis was undergone for the 67 children for which both the immediate and follow-up questionnaires were filled and the additional 6 children for which only the immediate questionnaire was filled (total = 73 children). There was no follow-up questionnaire completed without completing the immediate post-treatment questionnaire.

### Children’s initial characteristics

Median age of the 73 children included (33 females; 40 males) was 67 days (IQR = 112), with an age range spanning from 15 days to 5.5 years. Breastfeeding difficulties and preventative/wellness/no symptoms were the two most common reasons to seek care with both reported respectively by 30.3% (23/76) and 27.6% (21/76) of the legal guardians. Other reasons were neck pain (*n* = 8), plagiocephaly (*n* = 5), digestive issues (*n* = 4), reflux (*n* = 3), colic (*n* = 2), breech presentation (*n* = 2), temporomandibular joint dysfunction (*n* = 2), discomfort lying on the back (*n* = 2), neck weakness (*n* = 1), difficulty with tummy time (*n* = 1), tension after vacuum use during birth (*n* = 1), otitis (*n* = 1), muscular tension on the back (*n* = 1), brachial plexus injury (*n* = 1), leg and pelvis hyperlaxity (*n* = 1) and leg inequality (*n* = 1). All children received at least one spinal mobilization modality during their appointment. Almost all children were treated at both the cervical and the thoracic/lumbopelvic region (94.5%, 69/73). From the remaining children, 2.7% (2/73) were only treated at the cervical region and 2.7% (2/73) at only the thoracic/lumbopelvic regions.

### AEs analysis

Overall, AEs were reported in 30.1% (22/73, 12 males and 10 females) of the children. AEs were mostly observed immediately after the treatment (72.7%, 16/22). Only 4 children reported an AE at follow-up and 2 children reported AEs at both time points. With the exception of one AE that was a worsened symptom, all other AEs were reported as new symptoms. Most commonly reported AEs were irritability/crying (50.0%, 11/22) and fatigue/tiredness (50.0%. 11/22), followed by discomfort/pain (9.1%, 2/22) and sleeping disorders (9.1%, 2/22). Each of the following AEs was reported by one child: bowel movement, otitis, nausea/vomiting, and stiffness. No serious AEs were reported. A detailed description of the reported AEs per child is presented in supplementary file 1.

**Fig. 4:**
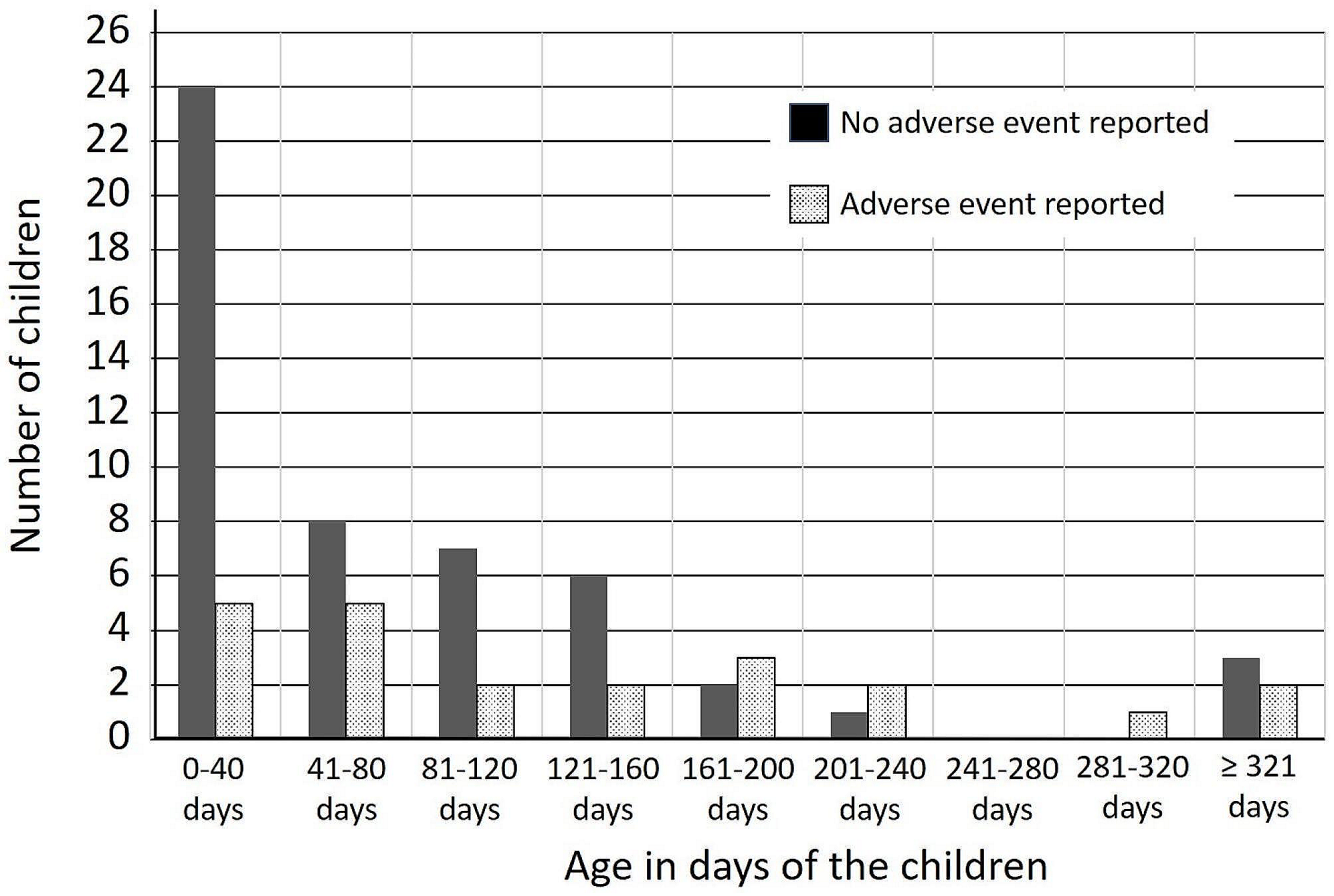
Number of children reporting or not reporting an adverse event (AE) based on age (in days)

Pearson’s chi-square test showed that the proportion of male (12/40) and female (10/33) children who sustained an AE was not significantly different: X^2^ (1, 73) = 0.182, *p* =.67. The number of children for which an AE was reported or not depending on age is shown in Fig. 4. Mann Whitney u test showed that the age of children reporting an AE (median = 88.5 days, IQR = 163) was statistically different from the age of children not showing an AE (median = 42 days, IQR = 98): *U*_(No AE = 51, AE = 22)_ = 761, z = 2.41, *p* =.02.

## Discussion

This study assessed the feasibility to conduct a pragmatic prospective study aiming to report AEs associated with manual therapies in children 0–5 years. Preliminary data regarding the AEs sustained by the children were also presented. Overall, 7 clinicians were successfully recruited and AEs were assessed in 73 children over the 33 weeks data collection period. Feasibility and preliminary data regarding AEs are discussed below.

### Feasibility data

Feasibility studies are conducted to determine whether a research methodology or intervention is appropriate for further testing and also for the sake of constant improvement [19]. Pohlman et al., 2021 [16] evaluated the feasibility of implementing an active-surveillance reporting system for AEs in adult patients seeking care at a chiropractic teaching clinic. Most of the burdens reported by Pohlman et al. [16] were not observed in the current study, which could be explained by the fact that this study was conducted in private clinics versus a teaching clinic. Additionally, Pohlman et al. [16] suggested putting the paper forms onto an electronic platform, to have better training on how to conduct the research, and to have an overall supervisor to implement the study. In the current study, the questionnaires were available on an electronic platform, all clinicians were explained study procedures during a meeting with one of the researchers, and frequent follow-ups were undergone with the clinicians to ensure proper implementation. Thandi et al. [20] identified that one major burden to clinicians’ engagement in practice-based research and learning networks is the lack of clinicians’ interest in the study topic. In the current study, 25.0% of the clinicians who view the Facebook post expressed their interest to participate, which is probably explained by targeting clinicians having a high proportion of their practice dedicated to pediatrics care. The first two participating clinicians mentioned that they greatly appreciated the easy access to communicate with the researcher if they had any concern during their data collection. Thandi et al. [20] also stated that regular communication between team members is a key factor for successful research. Future studies should be meticulously planned to address the substantial administrative workload associated with maintaining continuous communication between clinicians and the research team. This strategic approach aims to enhance the integration of research projects within private clinics, and it underscores the importance of potentially hiring research professionals to provide personalized support alongside clinicians.

### AE preliminary data

In the current study, AEs were reported in 30.1% of participating children which is a frequency closer to the ones reported in the adult population than in the pediatric population. For instance, Paanalahti et al. [13] reported that 37% of adult patients showed a minor or moderate AE following manual therapy. Similarly, Funabashi et al. [21] found an incidence rate of 23%, with higher risk of an AE in female patients aged 18 and older treated to the neck area. In the current study, differences between treated regions could not be analyzed given most of the children (94.5%) received manual therapies at multiple spinal regions. Noteworthy, some studies also reported incidence of AEs in adults up to 83% [22]. In contrast, the rapid review of Corso et al. [23] reported that the incidence of AEs in children ranges between 0.3 and 22.22%. More recently, Pohlman et al. [6], utilizing a paper version of the same active reporting system as in the current study to evaluate AEs associated with manual therapy in a pediatric population (*n* = 1894 treatments), reported a cumulative incidence of AEs, combining both immediate and up to one week post-treatment, at 8.8%. It’s worth noting, however, that their study encompassed patients up to 14 years old, in contrast to the current study, which only included patients aged 5 years or younger. While the authors did not provide the incidence rate for their sample of participants aged 5 years or less, AEs were more prevalent in this specific subset of pediatric patients. Additionally, Pohlman et al. [6] noted, in participants aged 5 years or less, the same three most common AEs of those reported in the current study: irritability/crying, pain/discomfort, and fatigue/tiredness. Further research comparing AE incidence within specific pediatric age ranges using a standardized methodology could help to clarify these discrepancies between studies.

Although it has been reported that women are more likely to report AEs following manual therapies than men [24, 25], the occurrence of an AE was not different based on the children’s sex. However, it could be hypothesized that the sex of the legal guardian could have a bigger incidence than the one of the children. This hypothesis remains to be evaluated in future investigations. It was also hypothesized that AE frequency will be higher in older children considering that it might be harder for the legal guardian to observe an AE in neonates or infants. Even if the results revealed that children showing an AEs were statistically significantly older, this result should be interpreted with caution due to the sample size.

### Strengths and limitations

The main strength of this study is the use of a reporting system that has been cross-culturally adapted from a validated English version [6]. While the French-Canadian version still requires validation, the use of this pre-validated system ensures consistency in reporting AEs and enhances standardization. Another strength is the recruitment success rate for both the clinicians and the children that support the feasibility to conduct a larger scale study.

The primary objective of this study was to assess the feasibility of the employed methods, rather than to draw inferential statistics on AEs. Given this context, the modest sample size was deliberately chosen to gauge the feasibility of the methods and was not designed to yield statistically significant outcomes regarding the frequency of AEs in children aged 5 years or younger. The results are presented primarily to guide future investigations and should be interpreted with caution. Furthermore, it’s important to highlight that data collection often occurred during the second treatment session of the child, influenced by the completion of forms by legal guardians. Consequently, it cannot be ruled out that some legal guardians may have discontinued care following the first treatment, particularly if more severe AEs were observed. In such cases, the current study would have missed these data. Additionally, it cannot be discounted that legal guardians may have informed the clinician at the onset of the second treatment about negative AEs observed after the first treatment, potentially prompting the clinicians to modify their care. Therefore, the methodology of the current study may result in an underestimation of AEs frequency. Moreover, the pragmatic approach, while enhancing alignment with clinical reality, limits associations to treatment visits. Therefore, a conclusive causal relationship between the treatment and the observed AEs cannot be established. While legal guardians were asked to report symptoms immediately following the treatment or in the days thereafter, they were not specifically questioned about whether they attributed the symptoms to the treatment (i.e., considered them as AEs) or to other factors, such as the discomfort of being manipulated by a stranger. Subsequent investigations should incorporate inquiries to legal guardians regarding their perceptions of the relationship between observed symptoms and the treatment itself. Finally, the external generalizability of the study may be influenced by clinicians administering only clinically indicated treatments to participants during the research. 

### Clinical perspectives

Clinicians must inform legal guardians of pediatric patients that symptoms, such as irritability, crying, fatigue and discomfort are frequent following a treatment involving manual therapies. However, investigations are still necessary to determine the relationship of these symptoms with treatment and the other factors related to the context of treatment.

## Conclusion

The study results support the feasibility to conduct a large-scale study evaluating AEs reported following chiropractic care in children of 5 years of less using the electronic SafetyNET reporting system. Findings suggest that targeting clinicians who have a marked interest for the treatment of children ensure clinicians’ engagement. The use of a reporting system available on an electronic platform resulted in a low attrition rate with at least 80% of the legal guardians completing at least one of the two follow-up questionnaires. Preliminary results showed that AEs were observed in about 30.1% of children and that irritability/crying and fatigue/tiredness were the most common AEs. Further patient-safety research is necessary in the pediatric population to properly inform legal guardians and clinicians on the potential AEs associated with manual therapies and thus providing a more fully inform consent to care.

### Electronic supplementary material

Below is the link to the electronic supplementary material.

Supplementary Material 1

## Data Availability

Data and materials will be provided on request to the corresponding author.

## Abbreviations

AE: Adverse event
CAM: Complementary and alternative medicine
REDCap: Research electronic data capture
ADHD: Attention deficit/hyperactivity disorder
UQTR: Université du Québec à Trois-Rivières
DICCP: Diplomate in clinical chiropractic pediatrics
ICA: International chiropractor association
