# Supplementary material for: Assessing adverse events associated with chiropractic care in preschool pediatric population: a feasibility study
Source: Chiropr Man Therap. 2024 Mar 13;32:9. doi: 10.1186/s12998-024-00529-0 (PMC10938841; doi:10.1186/s12998-024-00529-0)
Supplement: Supplementary file 1 — Supplementary Material 1 [file 12998_2024_529_MOESM1_ESM.docx]

**Supplementary file 1. Adverse events (AEs) reported by legal guardians immediately following the treatment and at follow-up (total = 22 children)**

| Participant | Age* | Sex | AEs reported immediately following the treatment | | AEs reported at follow-up | |
| --- | --- | --- | --- | --- | --- | --- |
|  |  |  | New symptom(s) | Worsened symptom(s) | New symptom(s) | Worsened symptom(s) |
| 1 | 21d | M | Fatigue/tiredness | -- | -- | -- |
| 2 | 26d | M | Irritability/crying | -- | -- | -- |
| 3 | 15d | M | Fatigue/tiredness | -- | -- | -- |
| 4 | ≈5m | F | Fatigue/tiredness | -- | -- | -- |
| 5 | ≈6m | M | bowel movement (defecated during the treatment) | -- | Irritability/crying, fatigue/tiredness | -- |
| 6 | ≈1m | F | -- | -- | Sleeping disorders | -- |
| 7 | 2.5m | F | Fatigue/tiredness | -- | -- | -- |
| 8 | ≈2y | F | Irritability/crying | -- | -- | -- |
| 9 | ≈6m | M | -- | -- | Irritability/crying | -- |
| 10 | ≈2m | F | Fatigue/tiredness, irritability/crying | -- |  |  |
| 11 | 10m | M | Others: otitis | -- | Irritability/crying | -- |
| 12 | ≈1m | F | Fatigue/tiredness | -- | -- | -- |
| 13 | 5.5y | M | -- | Fatigue/tiredness | -- | -- |
| 14 | ≈1m | F | -- | -- | Discomfort/pain | -- |
| 15 | 3m | F | -- | -- | Irritability/crying, nausea/vomiting | -- |
| 16 | ≈1m | M | Discomfort/pain, fatigue/tiredness, sleeping disorders, irritability/crying | -- | -- | -- |
| 17 | 4m | M | Fatigue/tiredness | -- | -- | -- |
| 18 | ≈3m | M | Irritability/crying | -- | -- | -- |
| 19 | 7.5m | M | Irritability/crying | -- | -- | -- |
| 20 | 7.5m | F | Irritability/crying | -- | -- | -- |
| 21 | ≈2m | F | Irritability/crying | -- | -- | -- |
| 22 | ≈6m | M | Discomfort/pain, stiffness | -- | -- | -- |

* d = days; m = months; y = years
